# From inside the country that never closed down: A qualitative research study focusing on the patient experiences of care and rehabilitation after the first wave of COVID-19 in Sweden

**DOI:** 10.1177/20551029251400494

**Published:** 2025-11-26

**Authors:** Linda Swanson, Christine Jones, Susann Azzabi, Anne Söderlund, Lena Nordgren

**Affiliations:** 1 Faculty of Medicine, 8097 Uppsala University, Uppsala, Sweden; 2 Centre for Clinical Research Sörmland, Uppsala University SE-Sweden; 3 Department of Paramedical Science, Region Sörmland; 4 School of Health, Care and Social Welfare, 8177 Mälardalen University, Västerås, Sweden; 5 Department of Public Health and Caring Sciences, 8097 Uppsala University, Uppsala, Sweden

**Keywords:** COVID-19, rehabilitation, hospitalization, patient care, qualitative research, interviews

## Abstract

This study aimed to explore the challenges that patients faced when severely ill with COVID-19 and during their rehabilitation journeys following the first wave in Sweden. Eight patients that were treated in an intensive care unit were interviewed using semi-structured interviews. Three themes were generated through thematic analysis: “transition into illness” (with subthemes: underestimated severity, uncertainty and worry); “to be cared for in a hospital setting” (with subthemes: loss of responsibility, loss of memory and time, contradictory feelings of being hospitalized, physical impact as frustrating); and “after care: managing on your own” (with subthemes: appreciation for care, care gaps and insufficient care, compromised ability, mental health, and self-efficacy for self-managed rehabilitation and post-traumatic growth). The findings indicated that the Swedish open strategy may be beneficial in other countries as it facilitated post-traumatic growth and that there should be a structured rehabilitation strategy in place in case of future pandemics.

## Introduction

As of August 2025, there were more than 778 million confirmed cases of COVID-19 and over 7 million deaths (The World Health Organization, 2025). The clinical presentation varies, with shortness of breath, fatigue, muscle and joint pain, sleep disturbances, headache, and cognitive difficulties being among the most common symptoms (Raunkiaer et al., 2024). Although the clinical characteristics and treatment of COVID-19 are well studied and described, the long-term problems faced by survivors remain unknown (Guo et al., 2022). This is especially true for patients who were critically ill because sedation, mechanical ventilation, and extracorporeal membrane oxygenation (ECMO) can lead to paranoid ideations, detailed delirious memories, and patchy factual memories (Gershfeld-Litvin and Ressler, 2023). It is clear that full recovery is not the same as clinical recovery in COVID-19 as 70% of those discharged from hospital did not consider themselves to be fully recovered 5 months post discharge (Gerlis et al., 2022). Depression, anxiety, and cognitive impairments are common (30–40% at 1-, 3-, 6-, and 12-month follow-up), as is an increased rate of psychotic symptoms and PTSD (post-traumatic stress disorder) (Gershfeld-Litvin and Ressler, 2023).

There is, however, evidence of post-traumatic growth (PTG) in COVID-19, with some studies documenting PTG rates as high as 60% (Xie and Kim, 2022). PTG (first described by Tedeschi and Calhoun, 1996) can be descried as the perception of positive psychological changes following a traumatic situation. This is where an individual’s core beliefs are challenged by a traumatic event, requiring the individual to grow from adversity. PTG has five aspects: enhanced relationships, discovering new possibilities, personal strength, spiritual change, and appreciation of life (Li et al., 2020) and is achieved through the interplay of individual characteristics (e.g. personality, coping styles), sociocultural processes (e.g. social support) (Xie and Kim, 2022), and environmental factors (e.g. physical exercise, social interactions, and collective activities and rituals) (Collazo-Castiñeira et al., 2022). Specific factors related to COVID-19 include the diagnosis period, coping strategies, self-esteem, emotional control, and social support (Yan et al., 2021). Psychological interventions that have been found to encourage PTG in the context of COVID-19 include mindfulness-based cognitive therapy (MBCT), photography, expressive writing, a character strength-based intervention, group counselling, and a self-distancing intervention (Adawia et al., 2023). In addition, PTG also seems to improve with self-efficacy (i.e., an individual’s belief in their own ability to achieve positive outcomes by acting in a certain way, Bandura et al., 1997). This could be because self-efficacy allows individuals to adjust to new circumstances with an optimistic attitude rather than rigidity (Zhang et al., 2021). In relation to the COVID-19 pandemic, self-efficacy was found to be a protective factor for the development of mental health during periods of lockdown (Ruotolo et al., 2023). In addition, an observational study on the rehabilitation of a subsample of patients with post COVID-19 condition showed that, amongst other things, the patients significantly increased their self-efficacy (in reaching goals, maintaining lifestyle, solving problems, and learning new skills) on completion of rehabilitation (Kupferschmitt et al., 2023). Furthermore, in an investigation of the effects of rehabilitation, patients with post COVID-19 condition significantly increased their self-efficacy regarding their return to work compared with care as usual (Nerli et al., 2024).

While most Western countries introduced lockdowns to limit the spread of the virus, Sweden followed a voluntary and comparatively open approach. Sweden’s differing policy approach, which received both criticism and praise, may offers lessons for future pandemics. The director of the World Health Organization (WHO) Dr Mike Ryan points out that: “*If we are to reach a ‘new normal’, in many ways Sweden represents a future model*” (Pashakhanlou, 2022). A large number of studies have been published on rehabilitation needs post COVID-19, where the majority of these use a quantitative approach and only few qualitative approaches (e.g. Gerlis et al., 2022; Raunkiaer et al., 2024; Gershfeld-Litvin and Ressler, 2023; Guo et al., 2022; Ladds et al., 2020; Luker et al., 2023). Guo et al. (2022) highlight that it is important to fully understand the treatment needs of COVID-19 survivors to guide health preventative health efforts and to plan healthcare management systems for future pandemics. As qualitative studies are needed to hear the patient’s voice, the aim of this study is to explore how individuals that suffered from severe COVID-19 during the first wave of COVID-19 in Sweden experienced the course of their illness and rehabilitation journey as this can help to tailor rehabilitation programmes. It is important to develop a deeper understanding of how the COVID-19 pandemic affected individuals in Sweden if a more open approach is to be used in other countries in future pandemics.

## Aim

This study aims to describe patients’ experiences of hospitalization during the first wave of COVID-19 as well as their experiences during recovery.

## Methods

### Design and context

A qualitative research design was used to answer the research questions. Participants were recruited from a 200-bed local hospital in mid-Sweden where all subjects gave their informed consent for inclusion. The study was conducted in accordance with the Declaration of Helsinki. The Consolidated criteria for Reporting Qualitative studies (COREQ) 32-item checklist (Tong et al., 2007) was used to enhance the quality and then transparency of the research findings.

### Participants

A purposive sampling procedure was used to identify patients who had been in intense care for COVID-19 during the specific time period described as the first wave (i.e. early 2020). Eligible patients were identified through a local patient follow-up register. Inclusion criteria were: adults (18–65 years); discharged after hospitalization in 2020 due to moderate to severe COVID-19; having been on ventilator or high-flow oxygen treatment in intensive care or supplemental oxygen for at least 5 days during hospitalization; rehabilitation needs related to COVID-19; and employment or full ability to work before infection. The exclusion criterion was other main reason for hospitalization than COVID-19. All the included patients had to be able to understand and communicate in Swedish or be willing to communicate using an interpreter. Eligible patients received oral and written information about the study and were asked to provide written informed consent before inclusion. Eight patients (five men (62,5%) and three women (37,5%), aged 47–64 years, mean age 56 years) decided to participate, where the age and gender distribution is similar to the population severely affected by corona at the time (Mi et al., 2020).

### Data collection

Data were collected through individual qualitative interviews conducted in 2021. The interviews lasted for 20 min to 1 h, and they were conducted in an outpatient rehabilitation setting by the second and third authors, who are well experienced physiotherapists. A semi-structured interview guide was used. The interviews started with an open question: *Can you tell me about when you became ill?* The subsequent questions concerned hospitalization, rehabilitation both at the hospital and after discharge, patients’ recovery periods, work, and future plans.

### Data analysis

Data analysis was initiated by the first and last authors. The first author holds a Doctorate in Clinical Psychology with experience of qualitative research whilst the last author is an associate professor with extensive experience in qualitative research design. The interviews were thematically analyzed according to Clarke and Braun (2006). NVivo Academic for Win software (v.1.6.1) was used for the analysis.

The recordings were transcribed and read several times by the authors to familiarize themselves with the content. Meaningful units were identified and coded in relation to the research question. After the initial categorization, the two researchers critically discussed the coding and started to search for patterns in the data to identify underlying meanings. Themes related to the research questions were then constructed. Finally, all authors reviewed the evolving themes, which were then defined and named. The analysis was completed when consensus over the meanings was reached by the authors.

## Results

**Figure 1.**
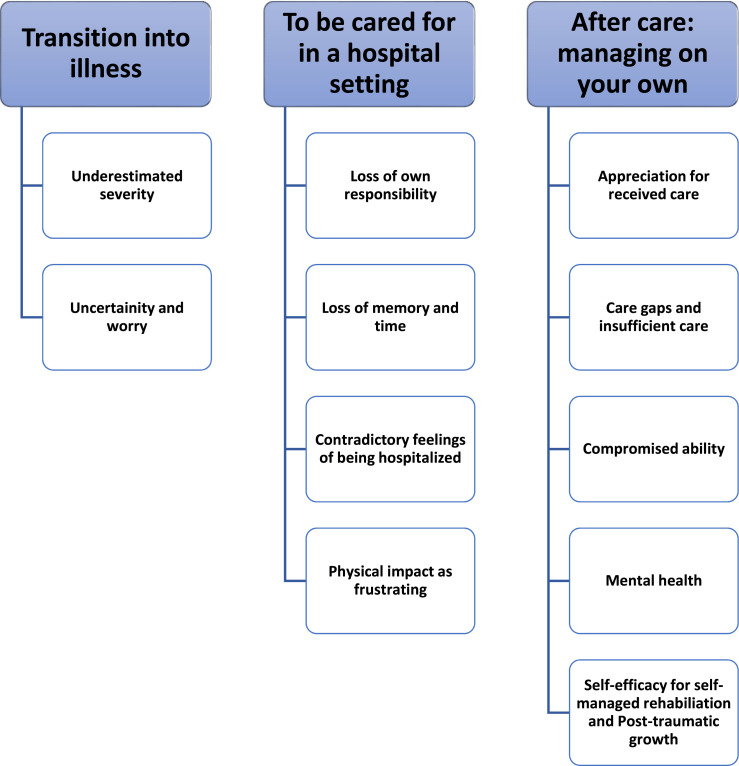
Main themes and subthemes.

The thematic analysis resulted in three main themes and 11 subthemes that described the informants’ experiences of hospitalization during the first wave of COVID-19 and their recovery period (Figure 1).

1. Transition into sickness

a. Underestimated severity

When the informants became ill, they did not initially understand that it was COVID-19. They described that they had noticed that they were unwell, but that they had failed to understand the severity of their symptoms and were oblivious to the risks of quick deterioration. The informants described a similar process in their social context, as they reported not being believed by relatives and/or when they contacted the healthcare service regarding their symptoms.

And then he said… stop being silly. He thought that I was joking (…) for God’s sake, I said, now you will have to get me to the hospital (…) He was just like, ah, get over it (…) and then I was very angry. I said that then you have two options: either you are driving me to the hospital and then I might survive, or you let me lie here to die. That's it. (R7)

b. Uncertainty and worry

Initially, there was uncertainty regarding contamination, spread, and testing. Some people tried to keep away from others or tested themselves. The presentation of illness varied from a few symptoms to many. Some patients experienced fast deterioration, while others were at home for up to 3 weeks before their condition needed medical care. The described symptoms were fever, difficulties with breathing, limited consciousness, stomach pain, sore throat, cough, illness, and poor appetite. Insight into being sick can bring feelings of fear or worry about dying. Simultaneously, some patients felt guilty about the risk of having contaminated others..

… Received my result on New Year’s Eve, and the main manager wasn’t there and it wasn’t taken seriously, so it spread. So I think, almost everyone had COVID there. And I had a very guilty conscience due to that and in the beginning I was also thinking that… people will die and that’s my fault. But it was only me who became this ill. (S5)

2. To be cared for in a hospital setting

a. Loss of responsibility

Receiving care for COVID-19 brought experiences of being vulnerable and dependent (for example, when patients had to be naked in front of others or needed help with intimate care). It was sometimes difficult to ask for help, as patients did not want to be perceived as demanding. However, patients also reported that admission entailed feelings of being taken care of and feeling safe.

Yeah. Well, right then I was actually just happy. They can do what they want, just as long as they care for me so I can breathe. That’s how it felt. I was… actually I wasn’t, right then, that afraid. That probably came later. Right then I was mostly focused or kind of, yeah… now I’m here. If anything happens then I’m here, mm. (S5)

Needing intensive care brought experiences of not having any other choice than to surrender oneself to the carers’ judgement and hope to survive and wake up.

I had no idea, what a respirator was. How it works, what happens to you and so on… there were so many questions. But it became like this: Yes, yes, you need to put me in a respirator, so then do it. That’s it… what should I say. There was nothing to argue about but if they considered it necessary, then I had to trust them that that was how it was. (R6)

b. Loss of memory and time

The interviewees described memory gaps or unclear memories of time at the hospital.

… and then I must have been very ill because I almost don’t remember anything. It’s afterwards that things come up that you remember well and so on. Yeah, they explained a bit, you have been lying here and it… and I couldn’t believe, when they woke me up… first of May. Then, I was clearer in my head, they just, yeah, now it’s first of May, and I just went: no, it’s April. I have only been lying here for a day or so. No, you have been lying down for twenty-one [days], almost a month. I just went no, it’s not possible… because it didn’t feel like I’d been lying… felt only like one and half, maximum two days. It never felt longer than that. (R2)

The information provided to the relatives was described as important and meaningful. This was partly for the relative’s own sake, for their comfort, but also so that they could share this with the affected relative to create a picture of the lost time. During the time in intense care, patients had dreams and hallucinations, which led to worry and an experience of being between dreams and reality.

Yeah, but it was… just then when you were in intensive care it was… before all the drugs had disappeared, then you were awake and then you fell asleep, then you were in the dream world again almost immediately. When you were awake again then you were in the normal world and then when you fell asleep lightly, kind of like… kind of like a surface that you disappeared under and then you are gone again. (R2)

c. Contradictory feelings of being hospitalized

Patients reported experiencing a lack of support from staff during their time in hospital. The interviewees gave the following examples: not knowing whether relatives had received information; feeling stupefied; being told to try to breath better when you could not breathe; perceiving that staff felt that you were demanding or that they were tired of you; being instructed to rest instead of trying to be up and active; not being given information about how the bed worked; not being able to borrow reading glasses; staff being inconsistent in their behavior and handling of technical equipment; and being told to lie quiet and still in bed.

I couldn’t move basically, eh… due to several reasons but mainly because of the muscles and I needed to go to the toilet, so I pressed the bell and no one comes or… trying then to get up on my own because I don’t want to wet the bed. And then you are kind of being told off, lie still and be… or lie still and be quiet kind of. That wasn’t kind of what I wanted then. Eh… and then the feeling of being trapped was reinforced. Then it became like a circle this, catch twenty-two. (R6)

Although the care setting was described as generally good, it was also described as boring due to a lack of activities. In some units, there was a TV in the person’s room. The patients spent time speaking to other residents or staff. However, the hospital stay could also be lonely without any contact or visits from relatives, which the patients found difficult. Food was remembered as good and well-presented. There was a wish to return home, but also gratitude for having survived. Being in proximity to others who were ill meant patients feared being contaminated in the hospital and becoming ill again. Hearing others’ distress is described as particularly difficult.

… but it was bloody hard down there, it was… though I was lying in my own room and you know… And it’s like it’s here, that there is a small hall and that door was also closed, eh… then I hear… there’s screams and there’s anxiety and so on. And it was… kind of, and you are lying there, you can’t do anything and you get to lie there and hear those, eh… felt, that it… people suffering. That was hard. I thought it was bloody hard and so on. (R7)

The residents were offered counselling to handle exposedness and vulnerability. Some patients found it difficult to accept the offer. Physiotherapy at the hospital offered patients hope of physical restoration and thus a faster return home. However, rehabilitation was often a struggle due to physical limitations caused by the illness even for patients that expressed a strong will to pursue physical rehabilitation. Furthermore, limited resources, such as a shortage of staff, hospital beds, and technical equipment (for example frames), meant that recovery and rehabilitation was further limited:

I wanted to get started with rehab so that I could move. And you can’t say that there was a rehab in X-town, there was just a walker /…/ but if I would have ended up here earlier, in rehab (I was upstairs then), then I think that I would have been well earlier. Then… we talk about a week here and there, but anyway. Just that feeling that damn, I want to get started and then getting “no, you need to rest”. (R6)

d. Physical impact as frustrating

Restoration and rehabilitation began during hospitalization. The patients described the physical limitations and changes that appeared during the hospital stay, including injuries caused by lying down for a long period of time, loss of muscle mass, decrease in strength, difficulties with coughing and mucus, general deterioration, weight loss, loss of smell and taste, and difficulties in dressing oneself due to unsteady hands. Physical changes meant fear and worry about the future, but also moments of hopelessness and resignation.

Then it was a bit like, just to start learning how to walk and write again and how to go the toilet on your own again (…) Yes, it took about 15 days. So it did… and about twenty-five days to walk a bit more. So I could get away from here, and it… one and a half week before you could learn how to eat food. Or, at least a week anyway, when I could eat food on my own. (I: Was it because lifting your arm was difficult?). Yes, difficult to lift. Yeah, left arm was totally gone. There were still problems with it then. (R2)

3. After care: managing on your own

a. Appreciation for received care

The participants reported that they appreciated the care they had been given. They felt heard, cared for, and seen in all steps of the rehabilitation process. They also expressed admiration and empathy towards the staff that they observed working hard despite a lack of resources, information, and previous experience of dealing with a pandemic. The participants also expressed that they had sufficient information about interacting with others when they were discharged.

IF: … when I came home they phoned… the doctor phoned after several days and asked how it was and, yes… so, yeah, there were several… then they phoned from primary care and asked how it was, and that felt good too. I felt kind of seen. (S5)

b. Care gaps and insufficient care

Although participants were generally impressed with the support they received during their time as an inpatient and through rehabilitation, some limitations in care were expressed in the interviews. The main shortcoming was the lack of help in making sense of their experiences through therapy, particularly as many experienced significant mental health issues relating to adjustment post discharge. In addition, the subjects felt that there was a lack of structured physical rehabilitation focusing on stamina and breathing once discharged. Instead, patients were left to implement a gradual increase in exercise on their own without any professional input or guidance. Participants also mentioned the lack of neuropsychological assessment/rehabilitation for the brain fog they experienced and felt that this would have facilitated the transition back to work. Patients felts that neuropsychological rehabilitation could have been conducted in a group setting to receive peer support. There was some frustration expressed around the lack of coordination between different healthcare agents (e.g. hospital staff and primary care/pharmacy) and externally (Swedish social insurance agency, Försäkringskassan), as well as a wish for more support with negotiation around practical and bureaucratic issues (e.g., medication, sick leave, and rehabilitation) where the participants were met with scepticism. The participants mentioned a significant amount of stress related to waiting times and finances. The participants acknowledged the difficulties faced by healthcare providers in tailoring services for different patient groups (e.g., elderly/stroke patients) at such an early stage in the pandemic. Some practical limitations were also acknowledged (e.g., support was often provided on the phone to limit patient contact, which sometimes felt insufficient, getting home from hospital without sufficient functional ability, data not being transferred into patients’ digital medical records).

IF: Well, you asked me if there was anything more important that I wanted to add. Yeah, I would have liked to speak to a neuropsychologist. I still haven’t. And that.. what would it lead to?, it could be…. I don’t know but maybe to something so that he can write something down. But, yeah… and then it is about this with the dreams and all that. Eh… I have processed a lot of shit on my own and can cope. But like I said, I think it is hard to fall asleep because I lie there and think about these things sometimes and so on. It’s not like I want sleeping pills or something like that… Anyway. But it could be good if one got to speak to this so called neuropsychologist. Eh….and that it happened through the state healthcare rather than I go to one privately. Because he has no connection to corona and it has to be somebody so that it becomes documented that this is how they feel over here and over here, if this is something that is pervading among others. (R7)

c. Compromised ability

Various remaining health issues were mentioned (e.g., no sensation in various body parts, loss of smell/taste, restricted movement, no physical strength, breathing difficulties, extreme physical/mental tiredness, and memory difficulties), which compromised patients’ capacity to take part in work and leisure activities. Individuals expressed fear and an inability to exert themselves due to these symptoms and a lack of belief and frustration around their current performance level (both physical and in terms of “brain fog”) compared to their pre-corona state. There was also an underlying concern about not being believed about how diffuse and varied their symptoms were.

IF: Yeah, it is horrendous, to be frank… It’s properly horrendous and I still think that it’s horrendous all that, eh… from being where you were and then end up here you know. And like this, no… and it is what it is too, I refuse to accept that… when you do these tests with the hands and things like that, yes but now, now you press more five more than what is normal for your age. And I don’t give a shit in that because… you aren’t allowed… My wife told me the other day, because she kind of thinks that, eh… I should be happy. I mean, I am happy that I am alive. It’s not that you know. But I became damned irritated the other day when I was going to… We have the bathroom cabinet on my side and I was going to pick up a tooth pick with this damned hand, and I can’t get it up because… Well, you can see why. No matter, some things… and it begins to… then I become so tired and I told her that, well then I was supposed to be happy for being alive and so on. That’s not it. You can be satisfied but you never stay satisfied. I compare myself to what I was before. (R7)

d. Mental Health

Participants reported a plethora of different feelings, thoughts, and processes related to their experiences. Many described an initial existential fear (which is very understandable as some cases were so severe that families were told to come to the hospital to say farewell) which persisted once discharged, especially when going to sleep. They also described more lingering anxiety in the early stages about not progressing and remaining highly dependent on others. There were also reports of a hopeless presentation, similar to a depressive state, when returning to everyday life and having to cope with demands put forward by the Swedish Social Insurance Agency and by the return to employment.

I: What has affected you most and being the most difficult during this period?

IF: Yeah, the most difficult… Well, from the beginning it was of course that you were so dependent when you were so sick. That you just… You can’t do anything on your own. It’s kind of hard work, not really knowing what is happening and so on, it is.

I: No. What do you remember of it?

IF: No, you get a bit afraid you know, because… will it be like this the entire life and that-

I: How was that feeling.

IF: Death anxiety. That’s what it was. Especially when you came home later. During the night. (S2)

e. Self-efficacy for self-managed rehabilitation and post-traumatic growth

Although many of the informants described that they would have valued more formal and structured rehabilitation, they also described a belief in their own ability to compensate for the lack of support. They described pushing themselves through mental and physical obstacles by being stubborn and persistent in designing their own rehabilitation (e.g., gradually improving the distances they were able to walk, run, swim, etc.).

IF: Then... well... then it’s different... one has a different type of sickness depending on how long you have been lying there. But I believe, get professional help and a bloody patience. You know, to try to get back. Because if you think that you can’t do this, then it’s difficult. (S4)

Individuals tried to make sense of their experiences and find meaning in them. Participants mentioned that they had begun to live a life closer to their underlying values, where they had more time for self-care and important relationships.

IF: If one should see something positive with this then it has turned out much better for me now than it maybe was before. Been quite a couch potato. And a bit more maybe also … this is for my sake. I am the one who should feel well from this (.)

IF: (…) Now I don’t mean to say that one should end up in the situation that I did, but it… for me it has been a reason to find something else in life-… that is, to become something better.

I: I see.

IF: And above all I also believe that one has actually…I only have one life, we all do. But I want to make the most of it.

I: Yes, exactly.

IF: And one needs to look after oneself.

I: Yes, that’s good.

IF: Sometimes I can feel… before, it was more for everyone else that…. You know, that’s how it is when you are family or friends. That is, you take one’s roles on so on. (R3)

## Discussion

This study explored individuals’ experience of severe COVID-19 and how they perceived the transition in and out of the acute phase and subsequent rehabilitation, during the first wave of COVID-19. It is important to fully understand how individuals suffering from severe COVID-19 in Sweden experienced their rehabilitation journey in order to guide preventative health efforts and plan healthcare management systems, especially if elements of the relatively open Swedish approach are replicated in other countries in future pandemics.

In the interviews, the patients stated that in the initial phase, they tended to underestimate the severity of their symptoms due to a disbelief in contracting COVID-19. They also reported that they experienced uncertainty and worry due to a lack of knowledge regarding prognosis and contamination once they had been diagnosed. The interviewees described their inpatient stay and the loss of personal-agency both as something positive as they felt safe and cared for but also as something negative due to feeling exposed and vulnerable. These findings are similar to previous qualitative research (Gershfeld-Litvin and Ressler, 2023) on Israelian survivors of COVID-19 post-mechanical ventilation and sedation who identified a theme of “an unexpected turn of events” in regard to becoming ill with two subthemes being “a state of surprise” and “loss of control.” Hence, it seems important to share reliable information with the public to encourage individuals to act early and avoid misconceptions. In addition, long-term rehabilitation interventions are needed due to the persistence of post-COVID symptoms (e.g. Cervino et al., 2020).

The interviewees also expressed feeling understimulated in the inpatient setting, a finding that was also identified in an Australian qualitative study (Luker et al., 2023). The interviewees reported feelings of loneliness due to a lack of time spent with staff whilst being an inpatient. Patients’ feelings of boredom and isolation can be alleviated by the recommendations in the Luker et al. (2023) study, which suggested increasing the staffing ratios for inpatients, social engagement from onsite staff, and support to staff to assist patients in using technology to be able to communicate with family members digitally (this is particularly important as social support is linked with PTG). It can therefore be considered important to prioritize time for staff to engage in social interactions in the future. This could also prevent unconstructive ward dynamics, as the patients experienced that staff perceived them as negative and demanding, which could be due to time constraints. The participants mentioned that they valued interactions with healthcare professionals because they felt listened to and heard (both during inpatient and outpatient experiences). In similar manner studies by Gershfeld-Litvin and Ressler (2023) and Luker et al. (2023), the participants said that the frequent updates given by the staff to relatives ware valuable as their families could help the patients account for time gaps during inpatient care in addition to calming and supporting the family at the time. This illustrates the importance of involving family members in the rehabilitation journey. We recommend starting the rehabilitation process earlier (preferably during in-patient care) as the participants, in agreement with previous qualitative research such as Luker et al. (2023), reported a readiness and desire for rehabilitation which was not met by the healthcare service. We found that when rehabilitation was provided, it was seen as meaningful, as it gave hope. It is therefore important to provide holistic rehabilitation early in the process. This could potentially be delivered efficiently through online interventions in the event of future pandemics, as discussed by Fiorillo et al. (2020).

The participants described various mental health difficulties (e.g., feelings of depression due to slow recovery and anxiety about the future), which mirrors previous research where use of ECMO, sedation, and mechanical ventilation led to similar psychological stress and symptoms (e.g., delirious and/or patchy factual memories, depression, PTSD, and anxiety) post-hospitalisation (Gershfeld-Litvin and Ressler, 2023). The patients identified various cognitive difficulties (e.g., fatigue and memory difficulties), which are also described in previous studies. These difficulties are also reported in subsequent waves of the pandemic despite shorter stays in the ICU (26 vs 9 days), which indicates that they might be caused by factors other than illness management (Möller et al., 2023).

This study identified a difference between inpatient and outpatient care, where the participants felt well cared for in the hospital setting but lonelier once discharged. Although this has been found in previous research on other health difficulties, the COVID-19 pandemic exacerbated this due to various factors (e.g., physical distancing, outpatient services closing, and early discharge from inpatient rehabilitation) (Wasilewski et al., 2022). The participants reported that they had a greater need for neuropsychological and physical assessment/rehabilitation in their home setting to understand and manage their limitations and that they needed therapeutic support to make sense of their traumatic experiences. They also expressed frustration over how badly the healthcare system interacted with external agents (which led to unnecessary financial stress) and worried that they would be questioned in regard to their compromised occupational ability due to their diffuse symptoms. As this was the first wave of COVID-19, there was very little rehabilitation due to the novelty of the disease. In a qualitative study by Raunkiaer et al. (2024), where a multidisciplinary (MDT) rehabilitation program designed for COVID-19 in Denmark was evaluated, individuals expressed a very different experience to the current sample as participants stated that they “*gained a completely different vocabulary and a much better understanding*” (p. 7). The participants expressed how the neuropsychological assessment proved that they were ill and gave them “*evidence, a physical thing which showed and measured that I am ill”* (p. 7), which, together with the rehabilitation and support from the MDT, helped in the process of returning to work. We also recommended MDT rehabilitation, which, as suggested by Wasilewski et al., (2022), could utilise telemedicine.

The participants reported a high degree of self-efficacy for self-managed rehabilitation with regard to their overall rehabilitation journey. They expressed that many aspects of their recovery had progressed well due to their own capabilities (e.g., stubbornness, persistence, and an imaginative approach to designing their own rehabilitation). This finding differs from qualitative studies from other parts of the world, where other factors were viewed as important for a positive outcome, such as religion (e.g. Gershfeld-Litvin and Ressler, 2023) and the national health system (e.g. Guo et al., 2022). This could be related to the fact that Sweden is an individualistic society rather than a collective society as collective societies tend to claim the joint effort of the network in managing the disease rather than the effort of the individual (Yu et al., 2021). This could also be the reason why stigma is more prominent in the Chinese study by Guo et al. (2022) than in the qualitative studies from Western societies, as higher levels of collectivism have been found to correlate with higher stigma which might be due to less diversity (Papadopoulos et al., 2013). It has been suggested that stigma during the COVID-19 pandemic might have been due to lack of trust in treatment, misinformation, administrative malfunction, and feelings of insecurity (Mahmud et al., 2021). Guo et al. (2022) suggest several actions to prevent stigma, such as providing inclusive and accurate information without stigmatizing terminology, and emphasizing the effectiveness of both prevention and treatment measures. This is important, as stigma can have detrimental effects on physical and psychological health, as well as making it difficult to control disease outbreak and management.

This study found clear evidence of PTG, as several participants explicitly mentioned that their priorities in life had shifted to focus more on relationships and living according to their values. This is also in line with previous research (Xie and Kim, 2022). As PTG is more evident in younger subjects and women, it is interesting that our sample (which mainly consisted of middle-aged men) still showed this pattern. This might have been due to the open Swedish approach, in which individuals could still participate in activities that have been shown to improve PTG (e.g., social interactions and physical exercise), or that the severity of the illness (which has been linked to higher PTG) created an increased need for adjustment and consolidation, or that the small sample size simply reveals individual coping mechanisms. The open Swedish approach might have also facilitated social support, which is believed to increase PTG through the reconstruction of positive beliefs (Collazo-Castiñeira et al., 2022) as well as facilitating cognitive processes (e.g. deliberate rumination) that are important for developing PTG (Henson et al., 2021).

## Limitations and strengths

A limitation of this study is the small sample size with eight participants included, which could be regarded as too few. However, the interviews revealed that the participants highlighted similar topics in their responses. Thus, even if we had had more participants, it is not certain that we would have gained different insights. Furthermore, some of the interviews were relatively short, potentially because the participant was too exhausted to talk longer or that the interviewers did not do enough to elicit more information from the interviewees. However, the collected data were relatively extensive and contributed to results that can be considered highly descriptive, making it possible to inform other similar contexts. Quotes from the interviews were used to establish trustworthiness. In addition, it could be suggested that interviews from healthcare providers could provide a valuable dimension to understanding the systemic challenges faced during rehabilitation. There is also a need for longer-term studies that track patient recovery and PTG over time. This would help in understanding the sustainability of self-managed rehabilitation and the long-term psychological impact.

## Conclusions

Rehabilitation in the event of future pandemics should consist of multidisciplinary and holistic rehabilitation where physical rehabilitation guides the body’s recovery, neuropsychological assessment identifies and validates cognitive difficulties to limit their impact when returning to work, and therapy helps process difficult experiences and fosters self-efficacy and post-traumatic growth.

## Data Availability

The data that support the findings of this study are available from the corresponding author, [L.S.], upon reasonable request.*
